# A Step Forward in Understanding the Expression of Classical Aquaporins in the Male Reproductive Tract: Study Findings in Cattle (*Bos taurus*)

**DOI:** 10.3390/ijms25147653

**Published:** 2024-07-12

**Authors:** Patrycja Oberska, Marta Grabowska, Marta Marynowska, Maciej Murawski, Dariusz Gączarzewicz, Andrzej Syczewski, Katarzyna Michałek

**Affiliations:** 1 Department of Physiology, Cytobiology and Proteomics, West Pomeranian University of Technology in Szczecin, Klemensa Janickiego 29, 71-270 Szczecin, Poland; patrycja.oberska@zut.edu.pl (P.O.); marta.marynowska@zut.edu.pl (M.M.); 2 Department of Histology and Developmental Biology, Pomeranian Medical University, Żołnierska 48, 71-210 Szczecin, Poland; marta.grabowska@pum.edu.pl; 3 Department of Nutrition, Animal Biotechnology and Fisheries, University of Agriculture, 24/28 Mickiewicza Avenue, 30-059 Cracow, Poland; rzmmuraw@cyf-kr.edu.pl; 4 Department of Animal Reproduction, Biotechnology and Environmental Hygiene, West Pomeranian University of Technology in Szczecin, Klemensa Janickiego 29, 71-270 Szczecin, Poland; dariusz.gaczarzewicz@zut.edu.pl; 5 Genetic and Animal Husbandry, Tomasza 40, 72-006 Szczecin, Poland; biuro@bydlo-as.pl

**Keywords:** water channel, male reproduction, animal development, testis, rete testis, efferent ducts, epididymis, vas deferens

## Abstract

Aquaporins (AQPs), also known as water channels, appear to be particularly promising in maintaining male reproductive potential. Therefore, this study aimed to determine the presence of classical AQPs in the bovine (*Bos taurus*) reproductive system and analyze changes in their expression with age using immunohistochemistry and Western blotting. Of the six classical AQPs, AQP0, AQP1, AQP4, AQP5 and AQP6 were detected, while AQP2 was absent. In the testis, AQP0 was visible in Leydig cells in selected animals, while AQP1 was found in myoid cells surrounding the seminiferous tubules of mature individuals. This characteristic expression patterns of AQP0, limited only to certain bulls, is difficult to explain unequivocally. It is possible that AQP0 expression in cattle is subject to individual variability or changes in response to specific physiological conditions. In the caput and corpus epididymis, AQP0 showed weak expression in epithelial cells of immature animals and stronger expression in basal and principal cells of reproductive bulls. In all animals, AQP1 was present on the apical surface of epithelial cells in the initial segment of the caput epididymis. AQP4, AQP5 and AQP6 were identified in principal and basal cells along the entire epididymis of reproductive bulls. The abundance of AQP4 and AQP6 increased from the caput to the cauda epididymis with the growth and development of the animals. In all males, AQP4, AQP5 and AQP6 were observed in epithelial cells of the vas deferens, and their expression in this section increased with age. In conclusion, the abundance and distribution of the classical AQPs in various cell types and parts of the male reproductive system indicate their crucial role in maintaining water homeostasis, which is essential for normal reproductive function in cattle.

## 1. Introduction

It is widely known that effective transport of water and solute in the individual segments of the male reproductive tract is a prerequisite for maintaining a unique microenvironment for a multi-step process of spermatogenesis, and the following concentration, maturation and storage of spermatozoa. Fluid movement already occurs within the testes, where Sertoli cells residing on the basal membrane of seminiferous tubules regulate the composition of tubular fluid. This fluid serves as the environment for spermatozoa development and facilitates their transport from the testis through the rete testis, efferent ducts, and epididymis and into the vas deferens [1]. A portion of the seminiferous tubular fluid also originates from differentiating germ cells. In fact, approximately 70% of the cell volume is osmotically eliminated from the cytoplasm during differentiation of round spermatids into elongated spermatids [2]. The composition of the fluid formed in the seminiferous tubules undergoes gradual modifications through concurrent processes of secretion and resorption of epithelial cells in successive segments of the male reproductive tract [3]. Undoubtedly, efficient fluid movement is facilitated by water channels located in male reproductive organs, also known as aquaporins (AQPs). AQPs belong to a family of small, hydrophobic, transmembrane proteins that facilitate the transport of water and a wide range of other substances, including glycerol, urea, carbon dioxide, ammonia, and hydrogen peroxide. In mammals, 13 members of the AQP family (AQP0-AQP12), located in many different cell types throughout the body, have been discovered thus far. The orthodox aquaporins, also referred to as classical aquaporins, constitute the largest subfamily of AQPs, comprising six members: AQP0, AQP1, AQP2, AQP4, AQP5, and AQP6 [4]. These proteins are considered to be primarily selective for water, although they may also facilitate transport of other small molecules. The exception is AQP2, which is selectively permeable solely to water [4]. The studies carried out to date, mainly in laboratory animals, have shown that at least five classical aquaporins, i.e., AQP0, AQP1, AQP2, AQP4 and AQP5, are present in the male reproductive system. AQP0 has been identified in rat Leydig and Sertoli cells [5]. AQP1 has been localized in the rete testis, efferent ducts, and epididymis in mice, while AQP2 has been observed in the vas deferens in mice and rats [6,7,8,9]. The presence of AQP4 has been found in the rat Sertoli cells, while AQP5 has been observed in the corpus and cauda regions of the rat epididymis [10,11].

Our previous study demonstrated that the expression and distribution patterns of aquaglyceroporins (AQP3, AQP7, and AQP9) within the reproductive organs of bulls varied in a tissue-specific and age-dependent manner [12]. Based on the results, it was concluded, among others, that AQP3 and AQP7 might play a significant role in the migration and proliferation of gonocytes, while all three aquaglyceroporins might be involved in the formation of microenviroment in the lumen of the epididymis. The present work is a continuation of our previous research in this area. So far, among the classical AQPs in cattle, only AQP1 in the epididymis and vas deferens of mature buffalo bulls has been analyzed [13]. In the context of the increase in male fertility disorders observed in recent years, the analysis of the location and expression of individual AQPs within the reproductive organs not only arouses great interest but also creates new and broad possibilities associated with the prediction of reproductive potential. Therefore, the aim of the present study was to determine the location of all classical aquaporins in the bovine male reproductive tract and analyze changes in their expression with age. Achieving this objective will enable testing the research hypothesis that classical aquaporins play a crucial role in maintaining fluid homeostasis within the male reproductive tract in cattle.

## 2. Results

### 2.1. Immunolocalization of Classical Aquaporins in Bovine Male Reproductive Tract

**Figure 1:**
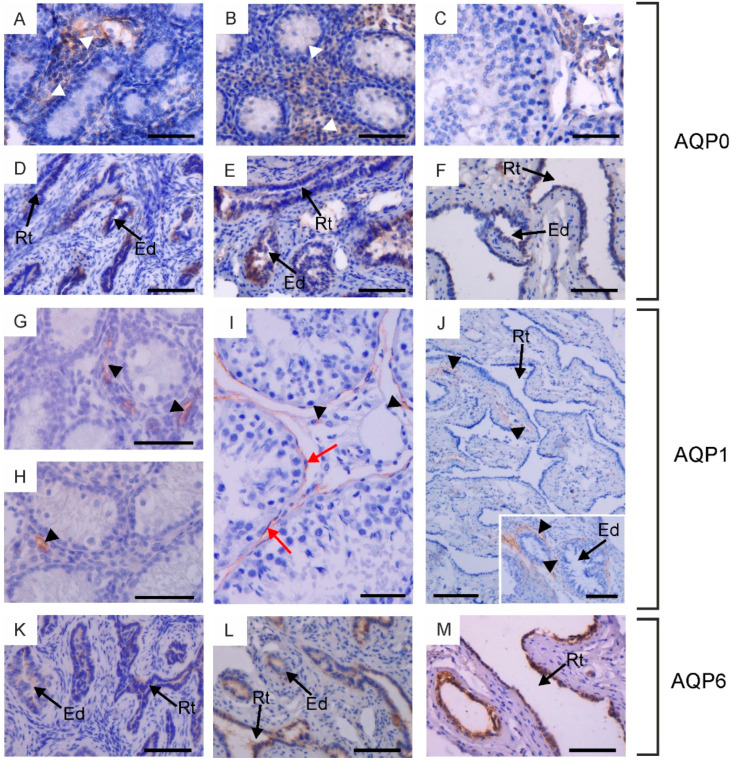
Immunohistochemical localization of AQP0 (**A**–**F**), AQP1 (**G**–**J**) and AQP6 (**K**–**M**) in the testis, rete testis and efferent ducts of calves (**A**,**D**,**G**,**K**), young bulls (**B**,**E**,**H**,**L**) and reproductive bulls (**C**,**F**,**I**,**J**,**M**). (**A**–**C**,**G**–**I**) Testis, (**D**–**F**,**J**–**M**) rete testis and efferent ducts. Black arrowheads, blood vessels; Ed, efferent ducts; Rt, rete testis; red arrows, peritubular myoid cells; white arrowheads, Leydig cells. Scale bar: (**A**–**M**), insert in (**J**) = 50 μm.

**Figure 2:**
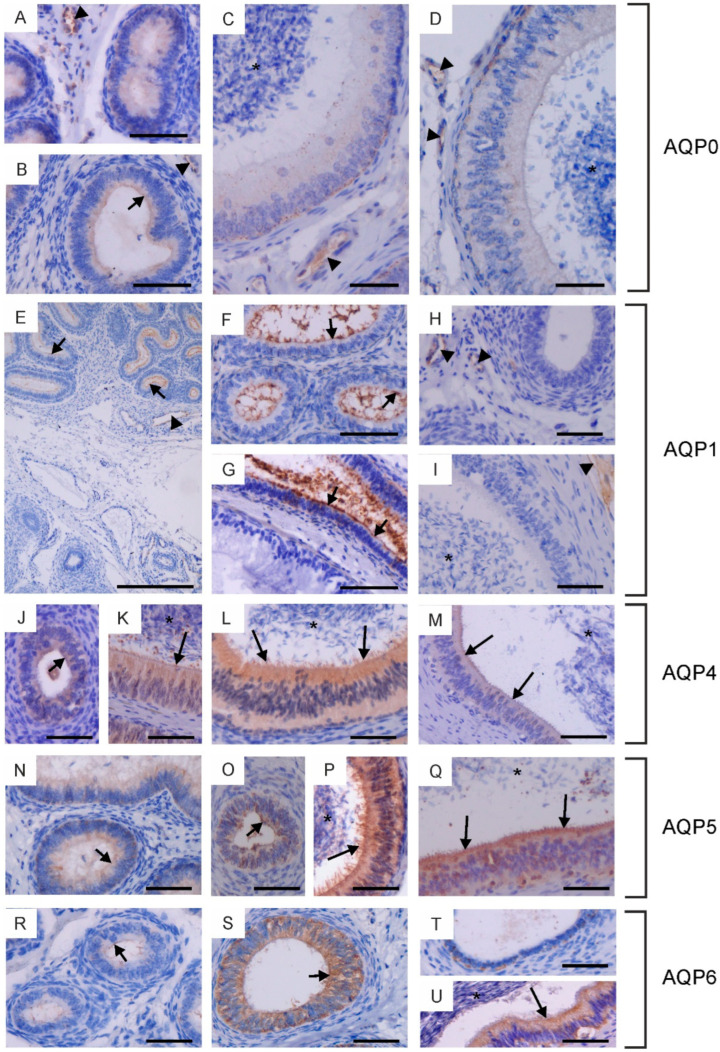
Immunohistochemical localization of AQP0 (**A**–**D**), AQP1 (**E**–**I**), AQP4 (**J**–**M**), AQP5 (**N**–**Q**) and AQP6 (**R**–**U**) in the epididymis of calves (**A**,**E**,**H**,**J**,**O**,**R**,**T**), young bulls (**B**,**F**,**N**,**S**) and reproductive bulls (**C**,**D**,**G**,**I**,**K**–**M**,**P**,**Q**,**U**). (**A**–**C**,**E**–**G**,**J**,**K**,**N**,**R**) Caput epididymis, (**D**,**H**,**L**,**O**,**P**,**S**) Corpus epididymis, (**I**,**M**,**Q**,**T**,**U**) Cauda epididymis. Asterisks, epididymal sperm; black arrowheads, blood vessels; black long arrows, stereocilia; black short arrows, apical plasma membrane of epithelial cells. Scale bar: (**A**–**U**) = 50 μm.

**Figure 3:**
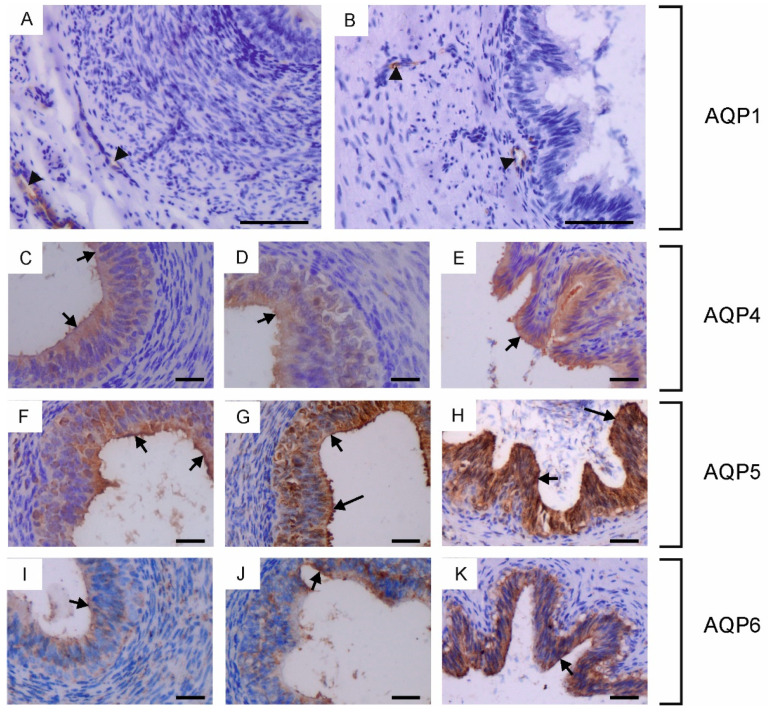
Immunohistochemical localization of AQP1 (**A**,**B**), AQP4 (**C**–**E**), AQP5 (**F**–**H**) and AQP6 (**I**–**K**) in the vas deferens of calves (**A**,**C**,**F**,**I**), young bulls (**D**,**G**,**J**) and reproductive bulls (**B**,**E**,**H**,**K**). (**A**–**K**) Vas deferens. Black arrowheads, blood vessels; black long arrows, stereocilia; black short arrows, apical plasma membrane of epithelial cells. Scale bar: (**A**–**K**) = 50 μm.

In the bovine male reproductive system, five of the six classical AQPs have been observed, namely AQP0, AQP1, AQP4, AQP5 and AQP6 (Figure 1, Figure 2 and Figure 3). The presence of AQP2 in reproductive organs was excluded in all animals studied. The semi-quantitative assessment of the total labeling intensity of identified AQPs was carried out using the adopted scale and is presented in Supplementary Tables S1–S4.

Within the testis, AQP0 and AQP1 were observed in the interstitial tissue across all animal groups. Weak expression of AQP0 was detected in the cytoplasm and cell membrane of Leydig cells (Figure 1A–C). It should be noted that this immunostaining pattern was observed only in half of the animals in each experimental group. AQP1 was visible in the endothelial cells of blood vessels (Figure 1G–I). AQP0 and AQP1 were not detected in the germ cells of the seminiferous tubules or in the Sertoli cells of calves, young and reproductive bulls. In sexually mature animals, weak to moderate immunoexpression of AQP1 was observed in the plasma membrane of peritubular myoid cells surrounding the seminiferous tubules (Figure 1I).

In the examined animals, immunoreactivity of AQP0, AQP1, and AQP6 was detected in the rete testis and efferent ducts. AQP0 and AQP6 were found in the cells lining the rete testis and efferent ducts (Figure 1D–F,K–M). These AQPs were predominantly localized in the cytoplasm and less frequently in the cell membrane. It should be emphasized that AQP0 was present exclusively in individuals where its expression was observed in Leydig cells. AQP1 was visible in blood vessels surrounding the rete testis and efferent ducts (Figure 1J). AQP6 expression in reproductively mature bulls in both of these structures was higher compared to sexually immature animals.

In the epididymis, AQP0, AQP1, AQP4, AQP5 and AQP6 were observed. AQP0 was present in the caput and corpus epididymis. In sexually immature animals, weak immunoexpression of this protein was recorded in the cytoplasm and plasma membrane (especially in its apical part) of the epithelial cells (Figure 2A,B). In reproductive bulls, AQP0 was observed in the cytoplasm and plasma membrane of basal and principal cells (Figure 2C,D). In both the caput and corpus epididymis, the abundance of AQP0 was greater in the basal cells compared to the principal cells. In all animals studied, AQP0 was also visible in blood vessels (Figure 2A–D). No AQP0 staining was detected in the cauda epididymis. In both sexually immature and mature animals, AQP1 was present in the initial segment of the caput epididymis (Figure 2E–G). This protein was observed at the apical surface of the epithelial cells. No AQP1 immunoreactivity was detected in the epithelium of the corpus and cauda epididymis. Along the entire epididymal duct, AQP1 was visible in the endothelium of blood vessels (Figure 2E,H,I). In calves and young bulls, weak to moderate expression of AQP4, AQP5, and AQP6 was predominantly observed in the cytoplasm, occasionally in the apical cell membrane of epithelial cells from the caput to the cauda of the epididymis (Figure 2J,N,O,R–T). In sexually mature individuals, AQP4, AQP5, and AQP6 were present in the cytoplasm and plasma membrane of principal and basal cells (Figure 2K–M,P,Q,U). In some cross-sections, AQP4, AQP5, and AQP6 were most abundant in the apical part of the epididymal epithelium (Figure 2L,P,Q,U). Immunoperoxidase labeling of AQP4, AQP5 and AQP6 was also detected in the stereocilia of principal cells (Figure 2K–M,P,Q,U). In reproductive bulls, the expression of AQP4 and AQP6 in the caput, corpus, and cauda epididymis was stronger than in calves and young bulls in the corresponding sections. AQP5 immunostaining decreased in the caput epididymis and increased in the corpus and cauda epididymis with growth and development of animals. No classical AQP was found in the epididymal sperm.

In the vas deferens, AQP1, AQP4, AQP5 and AQP6 expression was detected in all animals across the tested age groups. AQP1 was present in the blood vessels located around the vas deferens (Figure 3A,B), while AQP4, AQP5 and AQP6 were visible in the cytoplasm and plasma membrane of both principal and basal cells (Figure 3C–K). In young and reproductive bulls, AQP5 was also observed in the stereocilia of principal cells (Figure 3G,H). In the vas deferens, the immunoexpression of AQP4, AQP5 and AQP6 increased with the growth and development of the animals.

### 2.2. Immunoblotting of Classical Aquaporins in Bovine Male Reproductive System

**Figure 4:**
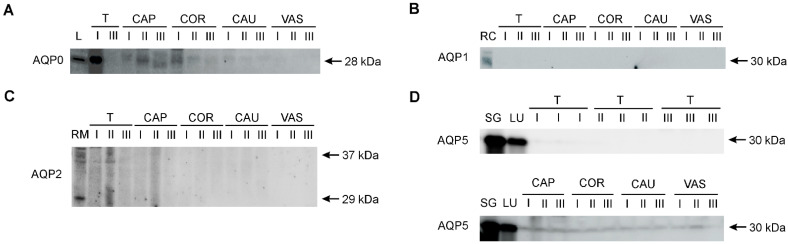
Representative Western blot images of AQP0 (**A**), AQP1 (**B**), AQP2 (**C**) and AQP5 (**D**) in the bovine male reproductive tract. I, calves; II, young bulls; III, reproductive bulls. T, testis; CAP, caput epididymis; COR, corpus epididymis; CAU, cauda epididymis; VAS, vas deferens. Bovine protein extracts were used as positive controls: L, eye lens (for AQP0); RC, renal cortex (for AQP1); RM, renal medulla (for AQP2); SG, parotid salivary glands (for AQP5); LU, lungs (for AQP5).

AQP0, AQP1, AQP2, and AQP5 were identified in the collected research material using the Western blot method (Figure 4). Control samples for individual AQPs confirmed the presence of AQP0 in the bovine lens, AQP1 in the renal cortex, AQP2 in the renal medulla, and AQP5 in the parotid salivary glands and lungs. The presence of AQP0 and AQP5 was confirmed in the male reproductive system. AQP0 was detected as a single band at 28 kDa in the testis, as well as caput and corpus epididymis (Figure 4A). No AQP0-specific signal was present in the cauda epididymis and vas deferens. AQP1 and AQP2 were only detected in a protein extract isolated from the bovine kidney (Figure 4B,C). In all animal age groups, AQP5 was detected as a single distinct band of 30 kDa in three regions of the epididymis (caput, corpus, and cauda) and in the vas deferens (Figure 4D). No AQP5 signal was observed in the testis. It was not possible to analyze the expression of bovine AQP4 and AQP6 using Western blotting with commercially available antibodies.

## 3. Discussion

In the present study, the cell- and tissue-specific distribution and expression of classical aquaporins were examined in various segments of the bovine male reproductive tract, along with their changes with growth and development of the animals. The testes are the main reproductive organs in males, responsible for producing spermatozoa and secreting sex hormones, primarily testosterone. Testicular tissue can be divided into two separate compartments, namely interstitial space and seminiferous tubules. Of all the aquaporins analyzed in the interstitial cells of the animals under study, only AQP0 was identified. This protein was detected in Leydig cells, but this distribution pattern was observed only in half of the animals in each experimental group. Published data indicate that AQP0 in Leydig cells has also been identified in other animal species, including horses and rats [5,14]. Additionally, AQP2 and AQP5 have also been observed in these steroidogenic cells in horses, while AQP1 has been identified in mice [14,15]. According to many authors, classical aquaporins located in Leydig cells contribute to maintaining water homeostasis between the extracellular and intracellular compartments [5,16]. Despite standardized study groups and identical conditions of IHC analysis, AQP0 was present only in some animals. Until now, both in our research and in studies by other authors, there have been no observations indicating that a specific aquaporin can be present or absent in certain individuals under physiological conditions in a specific organ. All tested animals were healthy and in good condition. We reanalyzed previously published data regarding AQP7 and AQP9 in Leydig cells in all animals studied [12], and no relationship was found between the expression of these aquaporins and the occurrence of AQP0. The presence or absence of AQP0 in bovine Leydig cells raises important questions about the potential impact of this protein on cell function. This issue undoubtedly requires further and more detailed studies.

AQP1 was also observed in the vascular endothelial cells within the testicular interstitium of all the examined individuals. Strong expression of this protein in blood vessels has been reported in various tissues and organs, such as the kidney, lung, skin, secretory glands, and skeletal muscle [17]. However, AQP1 was detected for the first time in the vasculature of the testes. This distribution suggests a role of this aquaporin in supporting water movement between the blood stream and the interstitial space, related to the functional specificity of the testes. In reproductive bulls, AQP1 was also observed in the cell membrane of peritubular myoid cells, which are the main cellular component of the walls of seminiferous tubules. In 1958, these cells were first discovered in rat testes and described as smooth muscle-like cells [18]. Currently, it is known that myoid cells are present in all mammals, although their organization differs between species [19]. In rats and mice, myoid cells are arranged in a single layer, while in humans, horses, and cattle, they exist in multiple layers [20,21]. To date, several functions of these cells have been established. One key role is their contractile activity, which is involved in the transport of non-motile spermatozoa and testicular fluid through the seminiferous tubules [22]. Myoid cells may also take part in the regulation of spermatogenesis by producing various hormones, cytokines and growth factors that modulate the function of Sertoli cells [23]. In addition, the peritubular myoid cell layer is an important contributor to the formation of the blood–testis barrier and structural integrity of seminiferous tubules [22]. Considering these facts, it is likely that AQP1 plays a significant role during the contraction of myoid cells, as it may facilitate water exchange between these cells and the interstitium to ensure volume changes during their contraction. Therefore, AQP1 may support the aforementioned expulsion of sperm cells through seminiferous tubules. It is worth noting that the testes of sexually immature animals do not yet produce spermatozoa, which may explain why the presence of this protein in peritubular myoid cells was recorded exclusively in reproductive bulls. In the animal species studied to date, among the classical aquaporins within the epithelium lining the seminiferous tubules, AQP0 and AQP4 have been observed in rat Sertoli cells, and AQP1 in bat spermatids [5,11,24]. Surprisingly, none of the aquaporins analyzed were detected in cattle in this structure. However, previous studies have indicated that AQP3 and AQP7 are present in germ cells, while AQP7 is present in Sertoli cells in these animals [12]. The absence of classical aquaporins in the bovine germinal epithelium is possibly compensated by the abundance of the aforementioned aquaglyceroporins. It should be noted that the presence of AQP8 and AQP11 in these cells cannot be excluded, as these aquaporins also facilitate membrane transport of water molecules.

After spermatozoa are released into the tubular lumen, the fluid created by Sertoli cells assists in the transport of the immature spermatozoa to the rete testis [25]. The rete testis is a network of small tubules located in the mediastinum of the testis, connecting the seminiferous tubules to the efferent ducts. The efferent ducts are responsible for reabsorbing up to 90% of the testicular luminal fluid, thereby increasing the concentration of spermatozoa and enabling interactions of their surface with the secretory products of the epididymal epithelial cells essential for sperm maturation [16]. In previous studies involving both of these structures, only AQP1 has been identified among the classical aquaporins. This protein was observed in the rete testis in dogs, rats and mice [8,26,27]. It is noteworthy that *Aqp1*-knockout mice showed only slight alterations in the rete testis as a result of obstruction of fluid reabsorption in the tubule lumen, and these animals were still fertile [28]. In efferent ducts, AQP1 has been reported in rats, mice, dogs, sheep, bats, and buffaloes [8,13,24,27,29,30]. In the test animals, AQP0 and AQP6 were observed within the epithelium of the rete testis and efferent ducts. The abundance of AQP0 in both of these structures was relatively low and was only observed in individuals where its presence was detected in Leydig cells. In the present study, it was found that the expression of AQP6 was higher in reproductive bulls. However, there is a lack of data in the available literature regarding the localization of this protein in the male reproductive tract in other animal species, which significantly complicates the interpretation of the results. However, it is known that unlike other aquaporins, AQP6 exhibits low water permeability and facilitates the transport of urea, glycerol, and anionic ions, particularly nitrate [31]. Given this information, it can be assumed that this aquaporin is involved primarily in the rapid movement of small solutes between the lumen and epithelial cells lining the rete testis and efferent ducts. Moreover, AQP1 in cattle has been detected in the endothelial cells of blood vessels in both of these structures. According to Badran and Hermo [29], the expression of this protein on vascular channels may mediate the removal of water from the intertubular space of these sections of the reproductive system.

**Figure 5:**
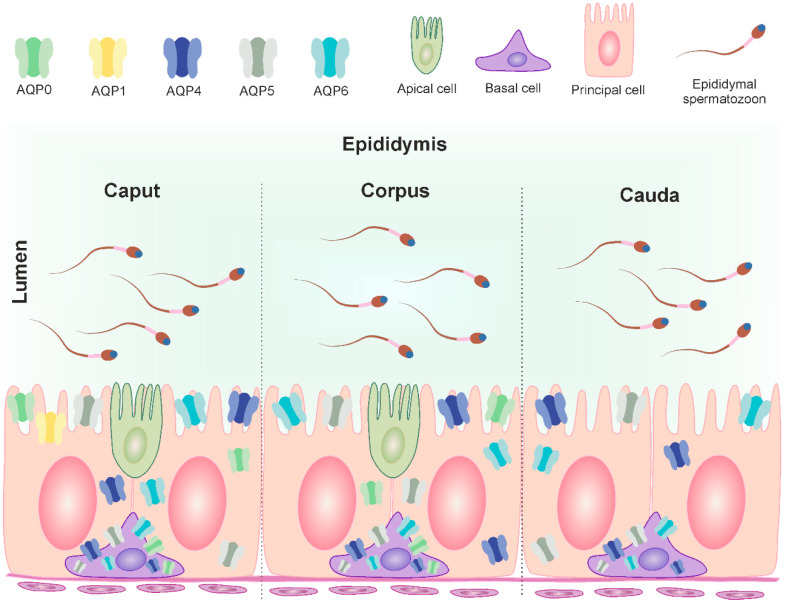
Distribution of AQP0, AQP1, AQP4, AQP5 and AQP6 in the three segments of the bovine epididymis (modified from Oberska et al. [12]). The figure illustrates the localization of mentioned AQPs in the cells of the mature epididymis and their subcellular compartments (plasma membrane, cytoplasm). The different classical aquaporins are identified in different colors. Caput epididymis: basal cell—AQP0, AQP4, AQP5 and AQP6; principal cell—AQP0, AQP1, AQP4, AQP5 and AQP6; apical cell—not determined. Corpus epididymis: basal cell—AQP0, AQP4, AQP5 and AQP6; principal cell—AQP0, AQP4, AQP5 and AQP6; apical cell—not determined. Cauda epididymis: basal cell—AQP4, AQP5 and AQP6; principal cell—AQP4, AQP5 and AQP6.

It is well established that testicular spermatozoa are immature and undergo maturational changes and acquire motility during their passage through the long highly convoluted tubule known as the epididymis [3]. This organ can be divided into three regions—caput, corpus and cauda—each with unique characteristics and function [32]. While previous studies have extensively examined classical aquaporins in the mature epididymis of sexually mature males, our study uniquely investigated their location and expression dynamics during animal growth and development. Of the classical aquaporins observed within the epididymis, AQP1 is currently the most comprehensively characterized. However, Yeste et al. [33] aptly pointed out that the distribution of this protein differs between species. In the epididymis of buffaloes, AQP1 has been observed only in the blood vessels [13]. In sheep, this aquaporin was found in the apical region of epithelial cells in the initial epididymal segment and in the microvilli of principal cells of the caput [30]. In mice, AQP1 has been observed on the membrane of smooth muscle cells surrounding the epididymal duct [8]. Previous studies have also demonstrated that in addition to AQP1, other AQPs, such as AQP0, AQP2, AQP4, and AQP5, are present in the epididymis of various animal species [9,10,14,34]. In the present experiment, five classical aquaporins have been identified in the bovine epididymis. However, it should be noted that not all of them were present along the entire epididymal duct (Figure 5). AQP1 was consistently found on the apical surface of epithelial cells in the initial segment of the caput epididymis and in the endothelium of blood vessels throughout the entire epididymis in all animals studied. The present results confirm earlier reports suggesting that AQP1 is primarily involved in fluid reabsorption in the proximal region of the epididymis [30]. In sexually immature animals, AQP0, AQP4, AQP5, and AQP6 were predominantly observed in the cytoplasm of epithelial cells. Specifically, AQP0 was detected in the caput and corpus epididymis, while AQP4, AQP5, and AQP6 were found in all three regions of the epididymis. The observed increase in their expression with the growth and development of animals indicates a progressive involvement of AQP0, AQP4, AQP5, and AQP6 in the formation of the epididymal microenvironment. In the current study, AQP0 was mainly observed in basal cells of reproductive bulls, and its expression appeared to decrease gradually along the successive segments of the epididymis, being most abundant in the caput, slightly less abundant in the corpus, and completely absent in the cauda. This protein was also reported in the blood vessels of the caput and corpus epididymis, which was consistent with previous observations in horses [14]. In sexually mature animals, AQP4, AQP5, and AQP6 were found in both principal and basal cells throughout the entire epididymis, indicating that these aquaporins participate in the reabsorption of a large amount of fluid within the lumen of this organ. These findings underscore the presence and coordinated function of multiple classical AQPs in various types and regions of the bovine epididymis, and their co-localization suggests their collective role in the transport of water and/or other small solutes to ensure optimal conditions for sperm concentration, maturation, protection, and storage.

The vas deferens, a part of the excurrent ducts, also plays a crucial role in transepithelial water reabsorption in the reproductive tract, maintaining the luminal environment essential for the next steps of spermatozoa maturation and subsequent storage [27,35]. Our findings revealed the absence of AQP0 and AQP2 in cattle in this region. However, AQP1 was found in the endothelial cells of blood vessels, while AQP4, AQP5, and AQP6 were detected in the epithelial cells in all examined animals. AQP1 expression in vessels surrounding the vas deferens was also recorded in buffaloes and dogs [13,27]. Among classical aquaporins identified in various animal species to date, AQP0 has been documented in the epithelium of the vas deferens in horses, AQP1 in mice and rats, and AQP2 in rats [7,8,14,26]. The presence of AQP1 in the vas deferens endothelium suggests its involvement in regulating fluid movement between vascular and interstitial spaces, similar to its role in other parts of the male reproductive system. However, the detection of three classical aquaporins in the principal and basal cells of the bovine vas deferens indicates significant movement of water and small solutes within this organ. Moreover, the abundance of AQP5 in the stereocilia of principal cells in young and reproductive bulls suggests an additional role of this protein in creating a unique microenvironment in the lumen of the vas deferens.

The location of and expression changes in individual AQPs in the male reproductive system are still not sufficiently known. Hence, a number of possibilities and various factors that may modify them should be taken into account. Among others, the increasing environmental pollution deserves attention, especially since male reproductive organs are particularly exposed to it. According to the latest research, environmental pollution has an impact on human semen and germ cells. It causes an increase in the concentration of immature cells, a decrease in the protamine/histone ratio, a reduced ability of sperm nuclear basic proteins, and a change in the copper/zinc ratio in sperm [36]. Marinaro et al. [37] analyzed the effects of exposure to single heavy metals (copper, nickel and cadmium) and their mixture on the reproductive system of marine invertebrate *M. galloprovincialis*. As a result of the conducted research, the authors of the mentioned studies found conformational alterations of protamine-like proteins that produced changes in their binding to DNA, which in turn resulted in atypical chromatin compaction of spermatozoa. In addition, a structural change in the male gonad was observed. There are no data in the available literature regarding the influence of various pollutants on AQPs; however, this influence cannot be ruled out. Further studies are needed to evaluate whether bioaccumulation of pollutants in the tissues of the male reproductive system affect the expression of AQPs.

Based on this and our previous research [12], a varied map of the location of AQPs was described in the testis. In all groups of animals, AQP0, AQP7 and AQP9 were found in the Leydig cells. Within the seminiferous tubules of immature animals, AQP3 and AQP7 were observed in the gonocytes. In the testicular tissue of the reproductive bulls, AQP1 was detected in the peritubular myoid cells, AQP3 in the spermatogonia and spermatocytes, and AQP7 in all germ cells and Sertoli cells. In the examined animals, AQP0, AQP3, AQP6 and AQP7 were found in the epithelial cells lining the rete testis and efferent ducts. In all studied individuals, AQP1 was visible in the apical surface of epithelial cells in the initial segment of the caput epididymis. In reproductive bulls, AQP0 was found in the caput and corpus epididymis, while AQP3, AQP4, AQP5, AQP6, AQP7 and AQP9 were observed along the entire epididymis. In sexually mature individuals, AQP0 and AQP3 were mainly visible in the basal cells. Immunoexpression of AQP4, AQP5, AQP6 and AQP7 was detected in both principal and basal cells. In turn, AQP9 immunostaining was noted in the stereocilia of the principal cells. In the bovine vas deferens, AQP3, AQP4, AQP5, AQP6, AQP7 and AQP9 were detected. These proteins share a similar labeling pattern in this organ in all animals tested. Mentioned AQPs were identified in the principal and basal cells, wherein immunostaining of AQP5 and AQP9 was also observed in the stereocilia. Within the male reproductive system, AQP1 was also found in the endothelial cells of the blood vessels.

Similarly to our previous study, certain methodological challenges were encountered during the experiments [12]. They pertained to the analysis of AQPs using Western blotting. With respect to AQP0 and AQP5, despite the clear detection of these proteins in controls, their expression was very low under the same conditions, even when the total protein quantity in samples from various segments of the male reproductive organs was doubled. Hence, it was not possible to reliably and credibly determine alterations in their expression. A challenge for modern science in the context of identifying and analyzing protein expression, including AQPs, is the search for alternative research methods that would enable their additional verification.

## 4. Materials and Methods

### 4.1. Animals and Tissue Collection

The experiments were conducted on the male reproductive organs obtained from 31 Polish, Holstein-Friesian, Black-and-White bulls (*Bos taurus*). Tissue samples were collected from three age groups of animals: (I) calves aged from 5 to 6 weeks (*n* = 10), (II) young bulls aged from 15 to 25 weeks (*n* = 10) and (III) reproductive bulls aged from 2 to 6 years (*n* = 11). Detailed information regarding the studied animals and their origin were described in a previous work [12]. The bulls from groups II and III were slaughtered according to the standard slaughterhouse procedures. After slaughter, the male gonads were immediately removed and dissected. Representative fragments of each testis, epididymis (caput, corpus and cauda regions) and vas deferens were collected and cut into small uniform pieces. Each fragment of the right reproductive organs was fixed in 10% buffered formalin, processed, embedded in paraffin blocks, cut into 2 μm-thick sections and subjected into immunohistochemical staining. Morphological and morphometric analyses of the studied material were conducted in a previous study [12]. Each representative region of the left reproductive organs was frozen in liquid nitrogen, stored at −80 °C and subsequently used for Western blot analysis.

### 4.2. Antibodies

All antibodies used in the present study and their dilution for immunohistochemistry and Western blot are shown in Supplementary Table S5. The specificity and antigenicity of commercially available antibodies against bovine AQP4, AQP5 and AQP6 have been proven in our previous work [38]. Homology between peptides sequence used for developing antibodies and amino acid sequences of bovine AQP0, AQP1 and AQP2 were defined on the protein blast database (https://blast.ncbi.nlm.nih.gov/Blast.cgi, accessed on 10 January 2024). Depending on the type of primary antibodies, labeling of the antigen–antibody complexes were visualized with the use of secondary polyclonal goat anti-rabbit or anti-mouse horseradish peroxidase-conjugated antibodies. Traditional methods of negative controls (i.e., omission of primary antibodies) were used in immunohistochemistry and Western blot.

### 4.3. Immunohistochemistry (IHC)

Immunohistochemistry was performed as previously described in detail by Michałek et al. [39]. After blocking endogenous peroxidase activity, sections were subjected to heat-induced antigen retrieval in citrate buffer at pH 6.0 (for AQP6) or in Tris-EGTA-buffer at pH 9.0 (for AQP0, AQP1, AQP2, AQP4 and AQP5). Sections were incubated overnight at 4 °C with the validated primary antibodies against bovine AQP0, AQP1, AQP2, AQP4, AQP5 and AQP6. After washing, samples were incubated for 1 h at room temperature with corresponding secondary antibodies. Immune reactions were visualized using 3.3′-diaminobenzidine (DAB) chromogen solution (Dako, Glostrup, Denmark, cat. no. K3468). All sections were analyzed using an Olympus BX43 light microscope (Olympus, Hamburg, Germany). Specificity of immunostaining was confirmed by following the above procedures, except that the primary antibodies were replaced with the same amount of IgG from bovine serum (Sigma Aldrich, Darmstad, Germany, cat. no. I5506; negative controls). In order to confirm the antigenicity of anti-AQP0, -AQP1 and -AQP2, positive controls with the use of bovine lens (for AQP0) and kidney (for AQP1 and AQP2) were additionally performed.

The immunohistochemical reactions were assessed by semiquantitative scoring based on labelling intensities as follows: no expression = “−”; weak = “+”; moderate = “++”; strong = “+++”.

### 4.4. Western Blot (WB)

The procedure was performed as described previously by Michałek and Grabowska [38]. Protein samples were separated on 12% Criterion TGX Stain-Free gels (Bio-Rad, Hercules, CA, USA; cat. no. 5678045) and transferred to PVDF membranes. After blocking, membranes were incubated overnight at 4 °C with primary antibodies against AQP0, AQP1, AQP2, AQP4 AQP5 and AQP6. The following day, membranes were washed and incubated at room temperature for 1 h with the corresponding secondary antibodies. Protein bands were visualized using an enhanced chemiluminescence system (Clarity^TM^ Western ECL Substrate, Bio-Rad, Hercules, CA, USA, cat. No. 170-5061), and blot images were acquired in a ChemiDoc MP imaging system (Bio-Rad, Hercules, CA, USA). For positive controls, bovine protein extracts were used, i.e., lens (for AQP0), renal cortex (for AQP1 and AQP4), renal medulla (for AQP2 and AQP6) and parotid salivary glands and lungs (for AQP5). The obtained images were recorded in a digital form and modified (autoscaling was applied and a representative band was cut out) using CorelDRAW (version 21.3.0.755, Corel Corporation, Ottawa, ON, Canada). Due to the low signal of the detected bands for AQP0 and AQP5, quantitative analysis of their optical density was not performed.

## 5. Conclusions

This study marks the first precise determination of the location and expression patterns of classical aquaporins throughout the bovine male reproductive system, from the testis to the vas deferens, across three animal age groups. These results have facilitated the development of a conceptual framework regarding the potential role of these proteins in maintaining water homeostasis in these crucial organs in male cattle. The presence of these aquaporins was recorded in various cell types and segments of the male reproductive tract, highlighting their importance in supporting proper reproductive function in cattle. The findings presented in this study represent a significant step forward in understanding the specific role of aquaporins in the physiology of the male reproductive system in mammals and provide a foundation for further research in this area. Combined with previously published data on the localization of aquaglyceroporins (AQP3, AQP7 and AQP9) in the same animals, these results represent another piece of the puzzle, forming a comprehensive picture of the distribution of these proteins in the bovine male reproductive system.

## Data Availability

The dataset that supports findings from this study is freely available at https://doi.org/10.34808/cjbc-yx88, accessed on 10 July 2024.

## Supplementary Materials

The following supporting information can be downloaded at https://www.mdpi.com/article/10.3390/ijms25147653/s1.
