# Supplementary material for: A Step Forward in Understanding the Expression of Classical Aquaporins in the Male Reproductive Tract: Study Findings in Cattle (Bos taurus)
Source: Int J Mol Sci. 2024 Jul 12;25(14):7653. doi: 10.3390/ijms25147653 (PMC11276675; doi:10.3390/ijms25147653)
Supplement: Supplementary file 1 [file ijms-25-07653-s001.zip › ijms-3105118-supplementary.pdf]

Supplementary material for:

A Step Forward in Understanding the Expression of Classical Aquaporins in the Male Reproductive Tract: Study Findings in Cattle (*Bos taurus*)

Patrycja Oberska, Marta Grabowska, Marta Marynowska, Maciej Murawski, Dariusz Gączarzewicz, Andrzej Syczewski, Katarzyna Michałek

Supplementary Table S1. Diagram showing staining intensity of AQP0 and AQP1 in the bovine testis in the three groups of animals.

	**AQP**	
	AQP0	AQP1
**Testis**		
**Calves**		
Gonocytes	-	-
Spermatogonia	-	-
Sertoli cells	-	-
Myoid cells	-	-
Leydig cells	+/-	-
**Young bulls**		
Gonocytes	-	-
Spermatogonia	-	-
Early developing spermatocytes	-	-
Sertoli cells	-	-
Myoid cells	-	-
Leydig cells	+/-	-
**Reproductive bulls**		
Spermatogonia	-	-
Spermatocytes	-	-
Round spermatids	-	-
Elongated spermatids	-	-
Residual bodies	-	-
Sertoli cells	-	-
Myoid cells	-	+/++
Leydig cells	+/-	-

Results are expressed by a scale: - = no expression; + = weak expression; ++ = moderate expression; +++ = strong expression.

Supplementary Table S2. Diagram showing staining intensity of AQP0 and AQP6 in the bovine rete testis and efferent ducts in the three groups of animals.

	**AQP**	
	AQP0	AQP6
	**Rete testis**	
**Calves**		
Epithelium	+/-	+
**Young bulls**		
Epithelium	+/-	+/++
**Reproductive bulls**		
Epithelium	+/-	++
	**Efferent ducts**	
**Calves**		
Epithelium	+/-	+
**Young bulls**		
Epithelium	+/-	+/++
**Reproductive bulls**		
Epithelium	+/-	++

Results are expressed by a scale: - = no expression; + = weak expression; ++ = moderate expression; +++ = strong expression.

Supplementary Table S3. Diagram showing staining intensity of AQP0, AQP1, AQP4, AQP5 and AQP6 in the bovine epididymis in the three groups of animals.

	**AQP**				
	AQP0	AQP1	AQP4	AQP5	AQP6
			**Caput epididymis**		
**Calves**					
Epithelial cells	+	++	+	++	+
**Young bulls**					
Epithelial cells	+	++	++	++	+/++
**Reproductive bulls**					
Basal cells	++	-	++	+	++
Principal cells	+	ND*	++	+	++
Apical cells	ND	ND	ND	ND	ND
Epididymal sperm	-	-	-	-	-
			**Corpus epididymis**		
**Calves**					
Epithelial cells	+	-	+	+/++	+
**Young bulls**					
Epithelial cells	+	-	+/++	+/++	++
**Reproductive bulls**					
Basal cells	+/++	-	+	++/+++	++/+++
Principal cells	+	-	++	++/+++	++/+++
Apical cells	ND	ND	ND	ND	ND
Epididymal sperm	-	-	-	-	-
			**Cauda epididymis**		
**Calves**					
Epithelial cells	-	-	+/++	++	+
**Young bulls**					
Epithelial cells	-	-	++	++	+/++
**Reproductive bulls**					
Basal cells	-	-	+	++/+++	++
Principal cells	-	-	+/++	++/+++	++
Epididymal sperm	-	-	-	-	-

Results are expressed by a scale: - = no expression; + = weak expression; ++ = moderate expression; +++ = strong expression. Abbreviation: ND, not determined. *In reproductive bulls, the intensity of AQP1 staining was not assessed in the initial section of the caput epididymis due to the limited number of samples.

Supplementary Table S4. Diagram showing staining intensity of AQP4, AQP5 and AQP6 in the bovine vas deferens in the three groups of animals.

	**AQP**		
	AQP4	AQP5	AQP6
**Vas deferens**			
**Calves**			
Basal cells	++	++	+/++
Principal cells	++	++	+/++
**Young bulls**			
Basal cells	++	++/+++	++
Principal cells	++	++/+++	++
**Reproductive bulls**			
Basal cells	++/+++	++/+++	++
Principal cells	++/+++	++/+++	++

Results are expressed by a scale: - = no expression; + = weak expression; ++ = moderate expression; +++ = strong expression.

Supplementary Table S5. Primary and secondary antibodies used for immunohistochemistry (IHC) and Western blot (WB).

Antibody	Dilution used for		Host	Type	Company	Cat. no.
	IHC	WB				
**Primary antibody**						
**anti-AQP0**	1:100	1:400	Mouse	Monoclonal	Santa Cruz Biotechnology	sc-376445
**anti-AQP1**	1:100	-	Mouse	Monoclonal	Santa Cruz Biotechnology	sc25287
	-	1:500	Mouse	Monoclonal	Abcam	ab9566
**anti-AQP2**	1:4000	1:250	Rabbit	Monoclonal	Abcam	ab199975
**anti-AQP4**	1:1000	N/A	Rabbit	Polyclonal	Novus	NBP1-87679
**anti-AQP5**	1:1000[1] 1:2000[2]	1:2500	Rabbit	Polyclonal	Abcam	ab78486
**anti-AQP6**	1:150	N/A	Rabbit	Polyclonal	Abcam	ab191061
**Secondary antibody**						
**Anti-rabbit IgG (HRP)**	1:200	1:2000	Goat	Polyclonal	Dako	P0448
**Anti-mouse IgG (HRP)**	1:2000	1:2000	Goat	Polyclonal	Abcam	ab205719

[1]Antibody dilution used for sections of the corpus and cauda epididymis, and vas deferens.

[2]Antibody dilution used for sections of the testis and caput epididymis.

Abbreviation: N/A – not applicable.

Note: In order to analyze the expression of AQP4 and AQP6 in the reproductive tract, the above mentioned commercially available anti-AQP4 and - AQP6 primary antibodies were tested. These antibodies were validated at various dilutions in WB. In addition, bovine protein extracts served as positive controls: renal cortex (for AQP4) and renal medulla (for AQP6). Unfortunately, the tested antibodies did not perform successfully in the WB analysis.
